# Development and testing of the reliability and validity of the adolescent haze related knowledge awareness assessment scale (AHRKAAS)

**DOI:** 10.1186/s12889-018-5638-8

**Published:** 2018-06-14

**Authors:** Hongzhe Dou, Yuejia Zhao, Yanhong Chen, Qingchun Zhao, Bo Xiao, Yan Wang, Yonghe Zhang, Zhiguo Chen, Jie Guo, Lingwei Tao

**Affiliations:** 1 Affiliated Hospital of Hebei University, No.212 Yuhua East Road, Baoding, 071000 China; 2 The NO.5 Hospital of Baoding, No.340 Ruixiang Street, Baoding, 071000 China; 3 College of Nursing, Hebei University, No.342 Yuhua East Road, Baoding, 071000 China; 4 School of Public Health, Capital Medical University, No.10 Xitoutiao, Youanmenwai, Beijing, 100069 China

**Keywords:** Haze, Adolescent, Reliability, Validity, Scale

## Abstract

**Background:**

Haze leads to many direct serious public health impacts. Understanding haze related knowledge can not only help adolescents organize health protection awareness to prevent the harmful effects that haze has on the body, but also promote their normal growth and development.

**Methods:**

By considering, as the theoretical basis, the reasons behind the formation of haze and the underlying mechanisms of the diseases that it causes, in addition to also investigating extensive literature references, our research team developed the Adolescent Haze Related Knowledge Awareness Assessment Scale (AHRKAAS-I). After 6 experts reviewed AHRKAAS-I, and 6 adolescents tested the scale, the research team further revised and improved AHRKAAS-I to form AHRKAAS-II. After which, researchers randomly selected 2 districts from the 20 districts of Baoding, and subsequently randomly selected 2 middle schools from these 2 districts. Conducting a stratified cluster sampling method, considering class as a unit, the research team randomly selected 22 classes. Finally, a total of 1100 adolescents were investigated and 1034 valid questionnaires were recovered. By analyzing the data of 1034 valid questionnaires, the researchers tested the reliability and validity of the scale and obtained the final scale (AHRKAAS).

**Results:**

AHRKAAS Cronbach’s α=0.923, content validity = 0.940, criterion validity = 0.444, and factor cumulative contribution rate = 66.178% by exploratory factor analysis. Using confirmatory factor analysis, Chi square value = 662.780, degrees of freedom = 242, Chi square value/degrees of freedom = 2.739, root-mean-square error of approximation = 0.049, goodness of fit index = 0.929, adjusted goodness of fit index = 0.905, comparative fit index = 0.964, normed fit index = 0.944, and Tueker-Lewis index = 0.955. AHRKAAS consisted of 25 items and 4 dimensions.

**Conclusion:**

AHRKAAS with a good reliability and validity can be used to assess the cognition level of haze related knowledge among the adolescents, help medical workers and coordinators in schools when conducting targeted behavior interventions. Furthermore, it can be used for health guidance for adolescents relating to the health prevention of haze.

## Background

Air pollution has become an important global issue, affecting the health of millions of people around the world [1]. Air pollution has multiple direct impacts on public health in China. People are aware of the correlation between air pollution and health [2, 3]. For the first time the Chinese Government initiated a National Plan on Air Pollution Control, which set out strict measures and goals to prevent and control air pollution in 2012. In September 2013, the Chinese Government promulgated the first National Action Plan on Air Pollution Prevention and Control(2013–17), which stated that by 2017 there would be significant and clear improvements to the quality of the air in China [4]. Short and long-term exposure to haze pollution are associated with a range of negative health outcomes, including respiratory diseases, cardiovascular and cerebrovascular diseases, mental health problems, lung cancer, and premature death [5]. As a form of manifestation of severe air pollution, haze has a harmful short-term acute affect in addition to long-term chronic effects on human health. These harmful consequences of haze can lead to long-term and far-reaching negative impacts on public health, especially for the health of children and adolescents [6–11]. Since adolescents are in a critical period of growth and development, their physical functions are not fully developed and hence are more susceptible to the impact of haze. This can result in damage to delicate physiological balance, which consequently has significant negative impacts to their health [1, 12–16]. Understanding haze related knowledge can help adolescents organize health protection awareness for the prevention of the harmful effects haze has on the body, reducing the incidence of related diseases, and promoting their normal growth and development, whilst also reducing the medical burden on the government, schools, society, and families [17]. Therefore, this study aimed to develop an Adolescent Haze Related Knowledge Awareness Assessment Scale (AHRKAAS) and test its reliability and validity to help medical workers in health care institutions, as well as school coordinators who conduct targeted behavior interventions and health guidance for adolescents in relation to the prevention of harmful effects of haze.

## Methods

### Development of AHRKAAS-I

By considering the reasons behind the formation of haze and the underlying mechanisms of the diseases that it causes as the theoretical basis [1, 16, 18–22], in addition to investigating extensive literature references, a pool of 42 items was collected. After analyzing and discussing, the research team retained 28 items and developed an initial scale (Adolescent Haze Related Knowledge Awareness Assessment Scale-I, AHRKAAS-I), which included 28 items and 3 dimensions. The three dimensions were named: Dimension 1, the cognition of influencing factors of the haze formation; Dimension 2, the cognition of haze harmful effects on the human body; Dimension 3, the cognition of haze health protection measures. All of the items were presented in a simple and easy-to-understand language in order to make the meaning of each item understandable for the adolescent population [23, 24].

### Development of AHRKAAS-II

Researchers invited 6 related experts from both the hospital and the medical university, including: two clinical doctors; two clinical nurses; and two full-time nursing teachers. Experts were responsible for assessing the content validity of AHRKAAS-I. The evaluation standard was set from 3 (strongly related) to 1 (not related). Based on the results from the expert reviews, 3 items of AHRKAAS-Iwere removed, which produced AHRKAAS-II. AHRKAAS-II included 25 items and 3 dimensions, with the unchanged names of each dimension. The scale used Likert 5-point method (5 = completely know; 4 = know most; 3 = moderately know; 2 = know a small part, 1 = don’t know). The total score of the scale was the sum of all of the items’ scores. The higher the score, the better the adolescent haze related knowledge awareness. Six adolescents were asked to complete AHRKAAS-II to test the wording and comprehension of the scale so that we could improve the wording and the statement expression of each item. All of the items were presented in easy-to-understand language to ensure that the meaning of each item was understandable for the adolescent population [23, 24]. The researchers then added an overall self-report item as the criterion to calculate the criterion validity of the scale: “Assuming that the full score of haze related knowledge is 100 points, how much do you think you are able to score?”

### Large sample test and development of the final AHRKAAS

From June 2015 to January 2016, researchers randomly selected 2 districts from the 20 districts of Baoding, China. Subsequently, from these 2 districts, 2 middle schools (one junior middle school, one senior middle school) were randomly selected. Conducting a stratified cluster sampling, considering class as a unit, the researchers randomly selected 5 first-grade classes, 5 s-grade classes, and 5 third-grade classes from the junior middle school (50 adolescents per class and a total of 750 adolescents). Furthermore, 3 first-grade classes, 2 s-grade classes, and 2 third-grade classes from the senior middle school (50 adolescents per class, with a total of 350 adolescents) were randomly selected. In total, the research team investigated 1100 adolescents between 11 to 20 years old (mean, 14.413 years old). Inclusion criteria: (1) Volunteered to participate in this research; (2) The adolescent has a normal understanding ability, no reading disabilities, or no intellectual disabilities. (3) Not suffering from mental diseases, brain diseases, or other serious illness. Sample size: Both exploratory factor analysis (EFA) and confirmatory factor analysis (CFA) was appropriately used when the hypothesized measurement model was evaluated. The sample size was at least 10–15 individuals per item for the factor analysis [25]. If the sample size was more than 20 individuals per item for the factor analysis, the results of factor analysis would be more stable and reliable [26]. Since this scale contained 25 items, the sample size of the large sample test should be more than 500. However, in order to increase the results stability and reliability, after comprehensively considering the feasibility of the study, we appropriately increased the number of samples. Therefore, for this study we planned to collect 1100 samples. Finally, a total of 1100 questionnaires were distributed. 19 adolescents did not complete the demographic characteristics questionnaire or the scale. 47 adolescents did not complete the scale. 1034 valid questionnaires were recovered. The valid recovery rate was 94%. By analyzing the data of 1034 valid questionnaires, the researcher tested the reliability and validity of the scale and ultimately produced the final version of the scale (AHRKAAS).

### Ethical consideration and survey method

The health and family planning commission of Hebei province approved this study (Permit Number: 20150072). The research team explained the purpose of this study to the middle school teaching management departments, as well as the adolescents of two middle schools. Parental/guardian written informed consent was obtained for participants under the age of 16 years old. As soon as consent was granted from the school leaders, parents and students, the researchers guided the adolescents about how to complete the questionnaires. The researchers distributed the questionnaires and subsequently recovered the questionnaires in the classrooms. The questionnaires were completed in an anonymous manner, and by using standardized language and unified instruction.

### Statistical analysis

We used the Epidata3.1 software to input the data into the computer twice, as well as to complete the consistency check. SPSS 17.0 and AMOS 17.0 software were used to analyze data. Researchers used descriptive statistics (frequency and percentage) to analyze the characteristics of the adolescents. Testing methods for reliability and validity of the scale: ①Exploratory factor analysis and confirmatory factor analysis were used to assess construct validity. ②Content validity index (CVI) was used to assess the content validity. ③The Pearson correlation coefficient was used to assess the criterion validity. ④The Cronbach’s α coefficient and the mean inter-item correlation coefficient (MIIC) were used to assess the reliability. The level of significance was set at *P* < 0.05.

## Results

### Characteristics of the large sample of adolescents

A total of 1100 adolescents were handed questionnaires, and 1034 valid questionnaires were returned. The valid recovery rate was 94%. The characteristics of the data of the large sample of adolescents are represented in detail in Table 1.

**Table 1:** Characteristics of the large sample of adolescents

Characteristics	Subjects	%
Gender		
Male	519	50.2
Female	515	49.8
Grade		
Junior high school	713	69.0
Senior high school	321	31.0
Race		
Han	999	96.6
Minority	35	3.4
Monthly cost (RMB)		
< 300	345	33.4
300~	541	52.3
600~	148	14.3
Do you have a religious faith?		
No	960	92.8
Yes	74	7.2
Place of residence		
Urban area	685	66.2
Rural area	349	33.8
Method of medical insurance		
Urban medical insurance	599	57.9
New rural cooperative medical system	309	29.9
Self-paying	126	12.2
Do you live with your family?		
Yes	967	93.5
No	67	6.5

Data representing characteristics of the large sample were presented as a frequency and percentage

### Testing of the reliability and validity

#### Construct validity

##### Exploratory factor analysis (EFA)

**Fig. 1:**
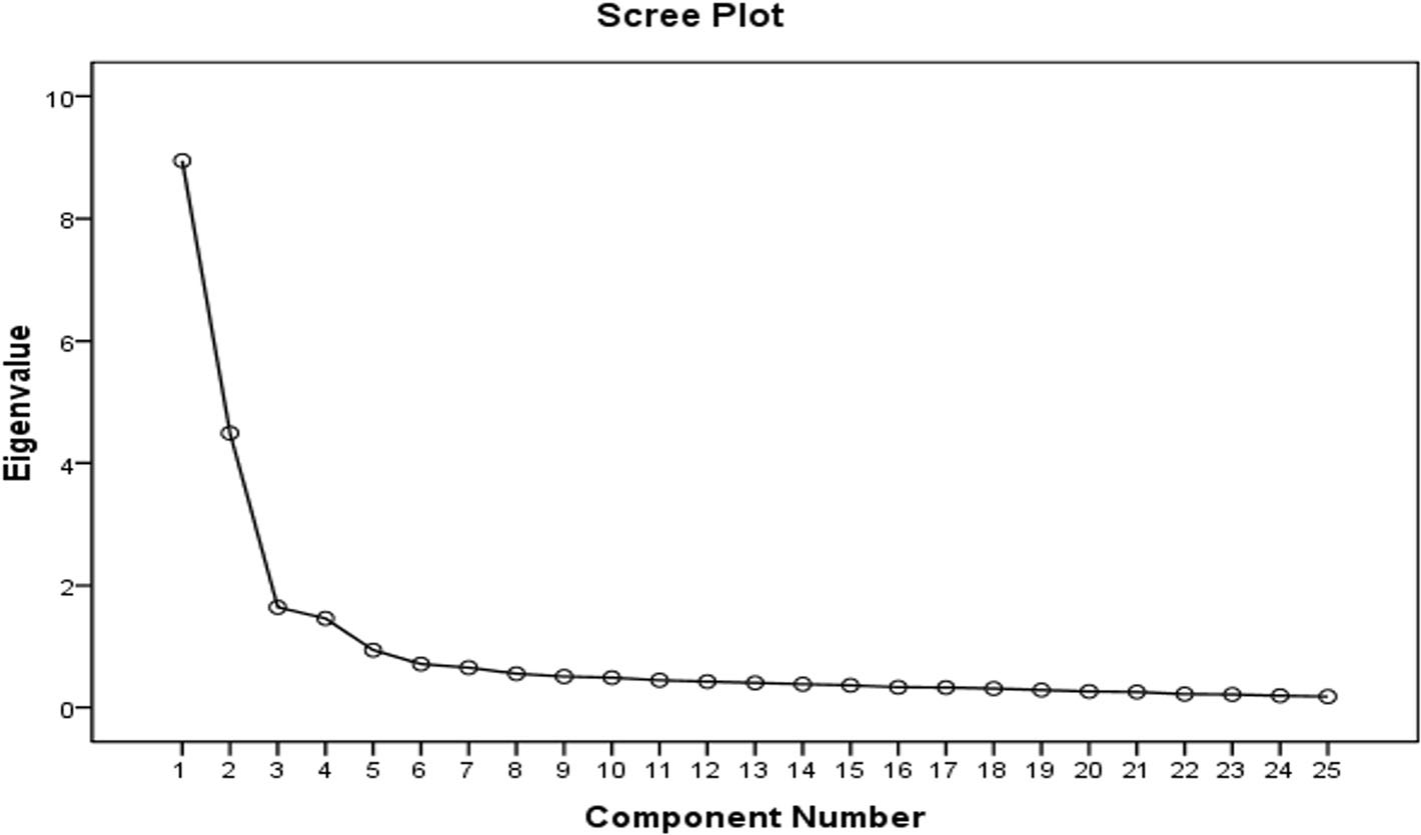
Scree plot of exploratory factor analysis

AHRKAAS-II was tested in the 1034 samples. The principal component analysis method (PCA) and the maximum variance orthogonal rotation method were used to carry out the exploratory factor analysis of AHRKAAS-II. The analysis results revealed that the Kaiser-Meyer-Olkin (KMO) value was 0.930 and the Bartlett sphericity test value was 16,954.675 (df = 300, *P* = 0.000). The results showed that data were suitable for factor analysis. Factor extraction was carried out under the condition of undefined factor number. Four factors (Eigenvalue> 1) were extracted and the cumulative variance contribution rate was 66.178%. The scree plot of AHRKAAS-IIfactor analysis showed an inflection point between the 4th and 5th factors. The scree plot of the factor analysis also showed that the 4-factor structure was suitable (Fig. 1). After comprehensive analysis, the final version of the scale (AHRKAAS) contained 4 factors and 25 items. The final 4 factors were renamed: Factor 1, the cognition of factitious factors of haze formation (7 items); Factor 2, the cognition of natural factors of haze formation (4 items); Factor 3, the cognition of haze harmful effects on the human body (9 items); Factor 4, the cognition of haze health protection measures (5 items). (Table 2). The detailed content of Adolescent Haze Related Knowledge Awareness Assessment Scale (AHRKAAS) is shown in Table 3, at the end of the result section.

**Table 2:** Rotated component matrix, eigenvalue and cumulative variance contribution rate

	Items	Factor 3	Factor 1	Factor 4	Factor 2
	Q1	_	0.812	_	_
	Q2	_	0.837	_	_
	Q3	_	0.831	_	_
	Q4	_	0.848	_	_
	Q5	_	0.746	_	_
	Q6	_	0.769	_	_
	Q7	_	0.757	_	_
	Q8	_	_	_	0.674
	Q9	_	_	_	0.714
	Q10	_	_	_	0.778
	Q11	_	_	_	0.732
	Q12	0.590	_	_	_
	Q13	0.730	_	_	_
	Q14	0.815	_	_	_
	Q15	0.819	_	_	_
	Q16	0.832	_	_	_
	Q17	0.837	_	_	_
	Q18	0.775	_	_	_
	Q19	0.732	_	_	_
	Q20	0.660	_	_	_
	Q21	_	_	0.685	_
	Q22	_	_	0.760	_
	Q23	_	_	0.785	_
	Q24	_	_	0.735	_
	Q25	_	_	0.628	_
Eigenvalue		5.634	5.263	3.034	2.614
Variance contribution rate (%)		22.535	21.053	12.134	10.456
Cumulative variance contribution rate (%)		22.535	43.588	55.722	66.178
Factor naming		The cognition of haze harmful effects on the human body	The cognition of factitious factors of haze formation	The cognition of haze health protection measures	The cognition of natural factors of haze formation

Factor 1, the cognition of factitious factors of haze formation; Factor 2, the cognition of natural factors of haze formation; Factor 3, the cognition of haze harmful effects on the human body; Factor 4, the cognition of haze health protection measures

This symbol ‘_’ indicates that values were less than 0.400. Suppress absolute values less than 0.400

**Table 3:** The content of adolescent haze related knowledge awareness assessment scale (AHRKAAS)

Dimensions	Items	Completely know 5	Know most 4	Moderately know 3	Know a small part 2	Don’t know 1
The cognition of factitious factors of haze formation	Q1. I know that factory emissions can cause haze.	5	4	3	2	1
	Q2. I know that burning agricultural straw can cause haze.	5	4	3	2	1
	Q3. I know that forest fires can cause haze.	5	4	3	2	1
	Q4. I know that burning garbage can cause haze.	5	4	3	2	1
	Q5. I know that coal-fired heating can cause haze.	5	4	3	2	1
	Q6. I know that dust produced from cars can cause haze.	5	4	3	2	1
	Q7. I know that automobile exhaust can cause haze.	5	4	3	2	1
The cognition of natural factors of haze formation	Q8. I know that when there is no wind, haze can be caused.	5	4	3	2	1
	Q9. I know that a decrease in rainfall can cause haze.	5	4	3	2	1
	Q10. I know that when the relative humidity of the air is high, haze can be caused.	5	4	3	2	1
	Q11. I know that when the temperature is low, haze can be caused.	5	4	3	2	1
The cognition of haze harmful effects on the human body	Q12. I know haze can cause pneumonia.	5	4	3	2	1
	Q13. I know that haze can cause lung cancer.	5	4	3	2	1
	Q14. I know that haze can cause the blood pressure to rise.	5	4	3	2	1
	Q15. I know that haze can cause heart disease.	5	4	3	2	1
	Q16. I know haze can cause dysfunction of the arteries.	5	4	3	2	1
	Q17. I know haze can cause dysfunction of the nervous system.	5	4	3	2	1
	Q18. I know haze can cause metabolic diseases.	5	4	3	2	1
	Q19. I know haze can cause reproductive dysfunction.	5	4	3	2	1
	Q20. I know the haze can cause allergic reactions in the body.	5	4	3	2	1
The cognition of haze health protection measures	Q21. I know that window opening time should be reduced in haze.	5	4	3	2	1
	Q22. I know that outdoor activities should be reduced in haze.	5	4	3	2	1
	Q23. I know that I should wear a protective mask in haze.	5	4	3	2	1
	Q24. I know that I should relax my mood in haze.	5	4	3	2	1
	Q25. I know that I should maintain enough sleep in haze.	5	4	3	2	1

##### Confirmatory factor analysis (CFA)

**Fig. 2:**
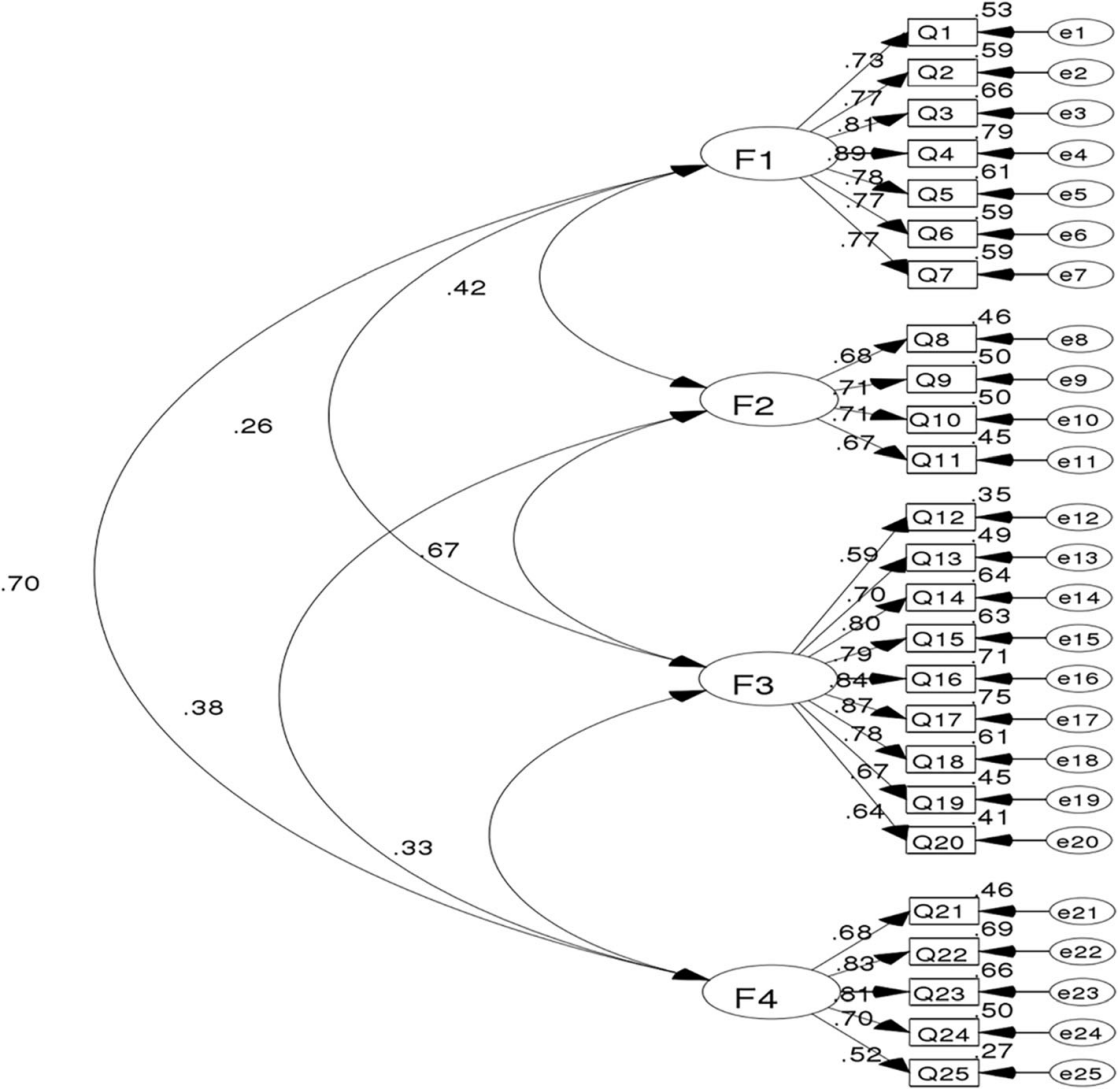
Standard path and parameter estimation of confirmatory factor analysis. F1, Factor 1, the cognition of factitious factors of haze formation; F2, Factor 2, the cognition of natural factors of haze formation; F3, Factor 3, the cognition of haze harmful effects on the human body; F4, Factor 4, the cognition of haze health protection measures. Suppress the correlation coefficient of the residual errors

In order to determine the best dimension structure of AHRKAAS, using the AMOS17.0 software, the researcher randomly selected a 70% sample size (724 samples) and used the maximum likelihood method to carry out the confirmatory factor analysis of the 25-item and 4-factor structure of AHRKAAS. Chi square value (χ2) was 662.780, degrees of freedom (df) was 242, Chi square value/degrees of freedom (χ2/df) was 2.739, root-mean-square error of approximation (RMSEA) was 0.049, goodness of fit index (GFI) was 0.929, adjusted goodness of fit index (AGFI) was 0.905, comparative fit index (CFI) was 0.964, normed fit index (NFI) was 0.944, Tueker-Lewis index (TLI) was 0.955 (Table 4). The standard path and parameter estimation of confirmatory factor analysis is shown in the Fig. 2.

**Table 4:** The results of confirmatory factor analysis

*χ* ^*2*^	df	*χ*^*2*^ /df	RMSEA	GFI	AGFI	CFI	NFI	TLI
662.780	242	2.739	0.049	0.929	0.905	0.964	0.944	0.955

χ2, Chi-square value; df, degrees of freedom; χ2 /df, Chi-square value/degrees of freedom; RMSEA, root-mean-square error of approximation; GFI, goodness of fit index; AGFI, adjusted goodness of fit index; CFI, comparative fit index; NFI, normed fit index; TLI, Tueker-Lewis index

#### Internal correlation test

The correlation coefficients among the factors of AHRKAAS were from 0.253 to 0.557 (*P* < 0.01). The correlation coefficients between the factors and the total scale of AHRKAAS were from 0.685 to 0.819 (*P* < 0.01). (Table 5).

**Table 5:** Correlation coefficients among the factors of AHRKAAS and between the factors and the total scale of AHRKAAS

Factor	Factor 2	Factor 3	Factor 4	AHRKAAS
Factor 1	0.353^**^	0.253^**^	0.557^**^	0.685^**^
Factor 2	_	0.543^**^	0.347^**^	0.722^**^
Factor 3	_	_	0.350^**^	0.819^**^
Factor 4	_	_	_	0.703^**^

Factor 1, the cognition of factitious factors of haze formation; Factor 2, the cognition of natural factors of haze formation; Factor 3, the cognition of haze harms on human body; Factor 4, the cognition of haze health protection measures. This symbol ‘_‘indicates no such correlation coefficient. ^**^P < 0.01

#### Content validity

According to the results of expert evaluation, the content validity index (CVI) of the scale was 0.940 and the CVI of each item ranged from 0.667 to 1.00. After improving the item wording and the statement expression, 6 adolescents reported that they could clearly understand the meaning of each item with no difficulty.

#### Criterion validity

Regarding the score of the overall self-report item relating to haze related knowledge as the criterion (“Assuming that the full score of haze related knowledge is 100 points, how much do you think you are able to score?”), researchers calculated the correlation coefficient between the total score of AHRKAAS (87.314 ± 20.227, mean ± SD) and the overall self-report item score (73.005 ± 16.098, mean ± SD). The correlation coefficient value was 0.444, *P* < 0.01.

#### Reliability

The mean inter-item correlation coefficients (MIIC) of each factor, as well as the whole AHRKAAS, ranged from 0.329 to 0.652. The Cronbach’s α coefficient of each factor ranged from 0.819 to 0.928. Cronbach’s α coefficient of the whole AHRKAAS was 0.923 (Table 6).

**Table 6:** The MIIC and the Cronbach’s α coefficient of each factor and the whole AHRKAAS

Factor	Number of Items	MIIC	Cronbach’s α
Factor 1	7	0.652	0.928
Factor 2	4	0.530	0.819
Factor 3	9	0.567	0.922
Factor 4	5	0.519	0.839
AHRKAAS	25	0.329	0.923

Factor 1, the cognition of factitious factors of haze formation; Factor 2, the cognition of natural factors of haze formation; Factor 3, the cognition of haze harms on human body; Factor 4, the cognition of haze health protection measures. MIIC, mean inter-item correlation coefficients

## Discussion

Globally, the health care model that regarded medical workers as having the leading role in patient care is gradually being transformed into a new modern health care model that places patients at the center of healthcare. In this newer model, individual health self-management capabilities are improved and both parties are able to jointly participate in decision relating to a patient’s healthcare [27, 28]. Publicity campaigns framed in the context of air pollution should be carried out to increase public awareness of the health risks and the corresponding personal prevention and protection measures. [5]. Some studies have highlighted the importance and urgency of air pollution control in China and have highlighted the protection of vulnerable populations [29, 30]. Therefore, by fully mobilizing the enthusiasm of adolescents, and by strengthening their awareness of health protection against haze, the harm caused by haze to adolescents may be reduced. At present, China’s medical system and policy guidelines predominantly focus on aspects of diagnosis, treatment, and care of specific diseases in hospitalized patients; however, investments in health protection and health care in locations such as communities, schools, and families are inadequate. Adolescents understanding of the harmfulness of haze is not enough, and their cognition level of haze related knowledge is low [31]. AHRKAAS developed in this study enables medical workers in health care institutions, as well as coordinators in schools, to quickly and effectively recognize the status of an adolescent’s haze related knowledge. These workers will be capable of conducting targeted behavioral interventions, as well as provide related health guidance for adolescents. This will help adolescents comprehend the concept and importance of haze health protection, which will lead to a lower incidence of related diseases. Ultimately this will result in a reduction of the medical burden placed on government bodies, schools, and families [32].

Both EFA and CFA are appropriately used when a hypothesized measurement model is evaluated [25]. The sample size should be at least 10–15 individuals per variable for the factor analysis [25]. The sample size in this study was large enough for the analysis. The factor analysis was suitable for this study as the KMO value (0.930) was greater than 0.6 and Bartlett’s test of sphericity (16,954.675, df = 300, *P* = 0.000) was significant [33]. The results indicated the data were suitable for factor analysis. EFA showed that 25 items loaded substantially onto 4 conceptually clear factors. Dimension 1(the cognition of influencing factors of the haze formation) in the AHRKAAS-II was divided into dimension 1(the cognition of factitious factors of haze formation) and dimension 2 (the cognition of natural factors of haze formation) in the final AHRKAAS version. The reason for the change of the dimensions was most likely because the cause of haze formation was composed of human factors and natural factors. These sources were different, hence we measured them from two separate dimensions. The 4-factor model produced a clearer and more accurate measurement of the structure of AHRKAAS. If the cumulative variance contribution rate was greater than 70%, it would have proven to be better [34]; however, the 66.178% of the AHRKAAS is also acceptable. The reason for this is that in some published literature relating to the development and testing of the reliability and validity of a scale, the cumulative variance contribution rate was also more than 60% and the validity of the scale was also acceptable [35]. As part of the CFA, the model goodness of fit was evaluated by RMSEA (< 0.08 acceptable), GFI (> 0.90 acceptable), AGFI (> 0.90 acceptable), CFI (> 0.90 acceptable), NFI (> 0.90 acceptable), TLI (> 0.90 acceptable) [24, 36]. All the results of CFA in this study met the above criteria. The results of CFA suggest that both the fit and the stability of the 4-factor model structure of AHRKAAS are satisfactory.

As part of the internal correlation test of AHRKAAS, the correlation coefficients between each factor and the total scale of AHRKAAS were from 0.685 to 0.819. This indicated that the internal correlation of the scale was good. The correlation coefficients between the various factors were from 0.253 to 0.557, indicating that there was a moderate correlation between the various factors [24]. The results revealed that there was a certain degree of correlation among the various factors, and that there were also some differences among the various factors. Thus, various factors could reflect different aspects of adolescent haze related knowledge, so that the four factors could comprehensively and effectively measure the cognition level of adolescent haze related knowledge.

Content validity refers to whether the items of the scale were able to identify the content and topic that we intended to measure. Each item content validity index of the scale represents the number of expert choices of 3 and 2 divided by the total number of experts. Total content validity index of the scale is the average of all of the items’ content validity indexes [24]. The CVI of the scale was 0.940, and the CVI of each item ranged from 0.667 to 1.00. This shows that AHRKAAS can reflect the variables measured. Each item is able to measure the correct content, and the AHRKAAS has good content validity.

Owing to the lack of a “golden criterion” for the measurement of the cognition level of adolescent haze related knowledge, measuring the criterion validity of AHRKAAS is a difficult task [34]. However, in the absence of a golden criterion, researchers from our team attempted to use the self-report method as a testing criterion. Researchers calculated the correlation coefficient between the total score of AHRKAAS and the overall self-report item score. The result revealed that the correlation coefficient value between the two variables was greater than 0.4; the correlation between them was moderate and the criterion validity of AHRKAAS was also acceptable [24].

The reliability of a scale can be determined by using Cronbach’s alpha and the mean inter-item correlation [37, 38]. The general criterion for a satisfactory internal consistency reliability is considered a Cronbach’s alpha of ≥0.7 [24]. In this study, Cronbach’s alpha values of each factor and the entire AHRKAAS were all greater than 0.8. If the mean inter-item correlation coefficient of a scale is greater than 0.3,the internal consistency reliability of scale is considered as acceptable [38]. MIIC values of each factor, and the entire AHRKAAS, were all greater than 0.3. Thus, through the above comprehensive analyses, AHRKAAS, in this study, proved good reliability.

## Limitations and future direction

Adolescents who participated in this study were recruited from the same city, Baoding city. Both the reliability and the validity of AHRKAAS were good among adolescents in the Baoding city; however, in future studies the reliability and validity of AHRKAAS should also be validated among adolescents in other cities that have different air quality, so that AHRKAAS can be more widely applied across the country. Due to limited research conditions, the scope of sampling is required to be further expanded in the future. Furthermore, AHRKAAS should be more widely applied and verified in a greater number of locations in the country, so that the scale can be further revised and improved. The test-retest reliability reflects the stability and consistency of AHRKAAS after a certain period of time [34]. Due to the limitation of the schools teaching plan and the limitation of the source of the objects in this study, the test-retest reliability of AHRKAAS was not tested. This should be verified in future research. Since a certain degree of cultural difference exists amongst different countries, it may have limited the generalizability of AHRKAAS. Thus, any effort to use this tool in other countries might require appropriate validation of AHRKAAS [34]. Further cross-cultural scale revision and scale validation are needed in order to develop an AHRKAAS that can be applied on an international scale.

## Conclusion

In summary, this study has rigorously developed and validated the AHRKAAS, which has proven to be reliable and valid. AHRKAAS can be used to assess the cognition level of haze related knowledge among the adolescents. Furthermore, it can help medical workers in health care institutions, as well as help school coordinators to conduct targeted behavioral interventions and provide health guidance for adolescents regarding prevention of the harmful effects of haze on health. Finally, AHRKAAS is a critical tool that helps adolescents establish a better awareness of this issue to enable them to promote their healthy growth.

## Abbreviations

AGFI: Adjusted goodness of fit index
AHRKAAS: Adolescent haze related knowledge awareness assessment scale
CFA: Confirmatory factor analysis
CFI: Comparative fit index
CVI: Content validity index
df: Degrees of freedom
EFA: Exploratory factor analysis
GFI: Goodness of fit index
KMO: Kaiser-Meyer-Olkin
MIIC: Mean inter-item correlation coefficients
NFI: Normed fit index
PCA: Principal component analysis
RMSEA: Root-mean-square error of approximation
TLI: Tueker-Lewis index
χ2 /df: Chi square value/degrees of freedom
χ2: Chi square value

## References

[1] Sierra-Vargas MP, Teran LM (2012). Air pollution: impact and prevention. Respirology.

[2] Qian X, Xu G, Li L, Shen Y, He T, Liang Y, Yang Z, Zhou WW, Xu J (2016). Knowledge and perceptions of air pollution in Ningbo, China. BMC Public Health.

[3] Liu X, Zhu H, Hu Y, Feng S, Chu Y, Wu Y, Wang C, Zhang Y, Yuan Z, Lu Y. Public's health risk awareness on urban air pollution in Chinese megacities: the cases of shanghai, Wuhan and Nanchang. Int J Environ Res Public Health. 2016;13(9):845. 10.3390/ijerph13090845 PMC5036678 27571088

[4] Chen Z, Wang JN, Ma GX, Zhang YS (2013). China tackles the health effects of air pollution. Lancet.

[5] Gao J, Woodward A, Vardoulakis S, Kovats S, Wilkinson P, Li L, Xu L, Li J, Yang J, Li J (2017). Haze, public health and mitigation measures in China: a review of the current evidence for further policy response. Sci Total Environ.

[6] Salameh P, Karaki C, Awada S, Rachidi S, Al Hajje A, Bawab W, Saleh N, Waked M (2015). Asthma, indoor and outdoor air pollution: a pilot study in Lebanese school teenagers. Rev Mal Respir.

[7] Dales RE, Cakmak S (2016). Does mental health status influence susceptibility to the physiologic effects of air pollution? A population based study of Canadian children. PLoS One.

[8] Dunea D, Iordache S, Pohoata A. Fine particulate matter in urban environments: a trigger of respiratory symptoms in sensitive children. Int J Environ Res Public Health. 2016;13(12):1246. 10.3390/ijerph13121246 PMC5201387 27983715

[9] Chen K, Glonek G, Hansen A, Williams S, Tuke J, Salter A, Bi P (2016). The effects of air pollution on asthma hospital admissions in Adelaide, South Australia, 2003-2013: time-series and case-crossover analyses. Clin Exp Allergy.

[10] Walton RT, Mudway IS, Dundas I, Marlin N, Koh LC, Aitlhadj L, Vulliamy T, Jamaludin JB, Wood HE, Barratt BM (2016). Air pollution, ethnicity and telomere length in East London schoolchildren: an observational study. Environ Int.

[11] Ding L, Zhu D, Peng D, Zhao Y (2017). Air pollution and asthma attacks in children: a case-crossover analysis in the city of Chongqing, China. Environ Pollut.

[12] Calderon-Garciduenas L, Vojdani A, Blaurock-Busch E, Busch Y, Friedle A, Franco-Lira M, Sarathi-Mukherjee P, Martinez-Aguirre X, Park SB, Torres-Jardon R (2015). Air pollution and children: neural and tight junction antibodies and combustion metals, the role of barrier breakdown and brain immunity in neurodegeneration. J Alzheimer's Dis.

[13] Poursafa P, Kelishadi R, Moattar F, Rafiee L, Amin MM, Lahijanzadeh A, Javanmard SH (2011). Genetic variation in the association of air pollutants with a biomarker of vascular injury in children and adolescents in Isfahan, Iran. J Res Med Sci.

[14] Stafoggia M, Cesaroni G, Galassi C, Badaloni C, Forastiere F (2014). Long-term health effects of air pollution: results of the European project ESCAPE. Recenti Prog Med.

[15] Islam T, Gauderman WJ, Berhane K, McConnell R, Avol E, Peters JM, Gilliland FD (2007). Relationship between air pollution, lung function and asthma in adolescents. Thorax.

[16] Provost EB, Chaumont A, Kicinski M, Cox B, Fierens F, Bernard A, Nawrot TS (2014). Serum levels of club cell secretory protein (Clara) and short- and long-term exposure to particulate air pollution in adolescents. Environ Int.

[17] Chanel O, Perez L, Kunzli N, Medina S, Aphekom G (2016). The hidden economic burden of air pollution-related morbidity: evidence from the Aphekom project. Eur J Health Econ.

[18] Dobreva ZG, Kostadinova GS, Popov BN, Petkov GS, Stanilova SA (2015). Proinflammatory and anti-inflammatory cytokines in adolescents from southeast Bulgarian cities with different levels of air pollution. Toxicol Ind Health.

[19] Poursafa P, Mansourian M, Motlagh ME, Ardalan G, Kelishadi R (2014). Is air quality index associated with cardiometabolic risk factors in adolescents? The CASPIAN-III study. Environ Res.

[20] Liao XN, Sun ZB, Tang YX, Pu WW, Li ZM, Lu B (2015). Meteorological mechanism for the formation of a serious pollution case in Beijing in the background of northerly flow at upper levels. Huan Jing Ke Xue.

[21] Liao XN, Zhang XL, Wang YC, Liu WD, Du J, Zhao LH (2014). Comparative analysis on meteorological condition for persistent haze cases in summer and winter in Beijing. Huan Jing Ke Xue.

[22] Gautam S, Yadav A, Tsai CJ, Kumar P (2016). A review on recent progress in observations, sources, classification and regulations of PM2.5 in Asian environments. Environ Sci Pollut Res Int.

[23] Billings-Gagliardi S, Mazor KM (2005). Development and validation of the stroke action test. Stroke.

[24] Zhao Q, Yang L, Zuo Q, Zhu X, Zhang X, Wu Y, Yang L, Gao W, Li M (2014). Instrument development and validation of the stroke pre-hospital delay behavior intention scale in a Chinese urban population. Health Qual Life Outcomes.

[25] Ekback M, Benzein E, Lindberg M, Arestedt K (2013). The Swedish version of the multidimensional scale of perceived social support (MSPSS)--a psychometric evaluation study in women with hirsutism and nursing students. Health Qual Life Outcomes.

[26] Xu Y (2011). Social survey design and data analysis - from topic to publication.

[27] Janiszewski D, O'Brian CA, Lipman RD (2015). Patient experience in a coordinated care model featuring diabetes self-management education integrated into the patient-centered medical home. Diabetes Educator.

[28] Blondon KS (2015). Patient attitudes about financial incentives for diabetes self-management: a survey. World J Diabetes.

[29] He T, Yang Z, Liu T, Shen Y, Fu X, Qian X, Zhang Y, Wang Y, Xu Z, Zhu S (2016). Ambient air pollution and years of life lost in Ningbo, China. Sci Rep.

[30] Li G, Huang J, Xu G, Pan X, Qian X, Xu J, Zhao Y, Zhang T, Liu Q, Guo X (2017). The short term burden of ambient fine particulate matter on chronic obstructive pulmonary disease in Ningbo, China. Environ Health.

[31] Jia P, Cai L (2014). Investigation on defense capability and information needs of haze / fog in Changping District,Beijing. Chin J Health Educ.

[32] Hedley AJ, McGhee SM, Barron B, Chau P, Chau J, Thach TQ, Wong TW, Loh C, Wong CM (2008). Air pollution: costs and paths to a solution in Hong Kong--understanding the connections among visibility, air pollution, and health costs in pursuit of accountability, environmental justice, and health protection. J Toxicol Environ Health Part A.

[33] Mills RJ, Young CA, Pallant JF, Tennant A (2010). Development of a patient reported outcome scale for fatigue in multiple sclerosis: the neurological fatigue index (NFI-MS). Health Qual Life Outcomes.

[34] Zhang Q, Huang F, Liu Z, Zhang N, Mahapatra T, Tang W, Lei Y, Dai Y, Tang S, Zhang J (2016). Cross-cultural validation of the high blood pressure health literacy scale in a Chinese community. PLoS One.

[35] Kourmousi N, Kounenou K, Tsitsas G, Yotsidi V, Merakou K, Barbouni A, Koutras V (2017). Active empathic listening scale (AELS): reliability and validity in a Nationwide sample of Greek educators. Soc Sci.

[36] Sun T, Zhao XW, Yang LB, Fan LH (2012). The impact of psychological capital on job embeddedness and job performance among nurses: a structural equation approach. J Adv Nurs.

[37] Reissmann DR, Benecke AW, Aarabi G, Sierwald I (2015). Development and validation of the German version of the orofacial esthetic scale. Clin Oral Investig.

[38] Komagamine Y, Kanazawa M, Kaiba Y, Sato Y, Minakuchi S (2014). Reliability and validity of a questionnaire for self-assessment of complete dentures. BMC Oral Health.

